# SIP metagenomics identifies uncultivated *Methylophilaceae* as dimethylsulphide degrading bacteria in soil and lake sediment

**DOI:** 10.1038/ismej.2015.37

**Published:** 2015-03-27

**Authors:** Özge Eyice, Motonobu Namura, Yin Chen, Andrew Mead, Siva Samavedam, Hendrik Schäfer

**Affiliations:** 1 School of Life Sciences, University of Warwick, Coventry, UK; 2 MOAC Doctoral Training Centre, University of Warwick, Coventry, UK

## Abstract

Dimethylsulphide (DMS) has an important role in the global sulphur cycle and atmospheric chemistry. Microorganisms using DMS as sole carbon, sulphur or energy source, contribute to the cycling of DMS in a wide variety of ecosystems. The diversity of microbial populations degrading DMS in terrestrial environments is poorly understood. Based on cultivation studies, a wide range of bacteria isolated from terrestrial ecosystems were shown to be able to degrade DMS, yet it remains unknown whether any of these have important roles *in situ*. In this study, we identified bacteria using DMS as a carbon and energy source in terrestrial environments, an agricultural soil and a lake sediment, by DNA stable isotope probing (SIP). Microbial communities involved in DMS degradation were analysed by denaturing gradient gel electrophoresis, high-throughput sequencing of SIP gradient fractions and metagenomic sequencing of phi29-amplified community DNA. Labelling patterns of time course SIP experiments identified members of the *Methylophilaceae* family, not previously implicated in DMS degradation, as dominant DMS-degrading populations in soil and lake sediment. *Thiobacillus* spp. were also detected in ^13^C-DNA from SIP incubations. Metagenomic sequencing also suggested involvement of *Methylophilaceae* in DMS degradation and further indicated shifts in the functional profile of the DMS-assimilating communities in line with methylotrophy and oxidation of inorganic sulphur compounds. Overall, these data suggest that unlike in the marine environment where gammaproteobacterial populations were identified by SIP as DMS degraders, betaproteobacterial *Methylophilaceae* may have a key role in DMS cycling in terrestrial environments.

## Introduction

Methylated sulphur compounds are significant intermediates in the global biogeochemical sulphur cycle owing to their role in transferring sulphur between the atmosphere, terrestrial and marine ecosystems (Lomans *et al.*, 1997; Bentley and Chasteen, 2004; Schäfer *et al.*, 2010). Dimethylsulphide (DMS), a volatile organic sulphur compound, provides the largest input of biogenic sulphur from the oceans into the atmosphere with approximately 21 Tg per year (Watts, 2000). Its oxidation products affect atmospheric chemistry and contribute to the atmospheric aerosol burden (Andreae, 1990). It has been suggested that DMS contributes to regulation of global climate by acting as a climate-cooling gas (Charlson *et al.*, 1987); although, whether this is indeed the case remains controversial (Quinn and Bates, 2011).

In the marine environment, breakdown of dimethylsulphoniopropionate (DMSP) is the major source for DMS (Johnston *et al.*, 2008). In contrast, the sources of DMS in terrestrial environments, including freshwater bodies, are diverse, including reduction of dimethylsulphoxide (Zinder and Brock, 1978), degradation of sulphur containing amino acids (Kadota and Ishida, 1972; Finster *et al.*, 1990; Kiene and Hines, 1995) and anaerobic degradation of methoxylated aromatic compounds (Bak *et al.*, 1992; Lomans *et al.*, 2001). Lomans *et al.* (1997) reported concentrations of DMS in freshwater sediments in the range of 1–44 nM, similar to, if not higher than typical DMS concentrations in the upper marine water column (Lana *et al.*, 2011). DMS is also emitted from a range of plant species including trees, cruciferous vegetables, lichens and wheat (Bending and Lincoln, 1999; Watts, 2000; Crespo *et al.*, 2012). Anthropogenic sources of DMS include the pulp and paper, dairy and brewery industries for instance (Keenan and Lindsay, 1968; Scarlata and Ebeler, 1999; Zhang *et al.*, 2006).

Annual DMS emission to the atmosphere from terrestrial ecosystems has been estimated at 3.8 Tg (Watts, 2000). As in marine systems, microbial metabolism of DMS is likely to have a major role in controlling DMS emissions by acting as a sink for DMS. Utilisation of DMS as a carbon and energy source by microorganisms has been well documented and a wide range of methylotrophic DMS degrading bacteria have been isolated from soils, biofilters, sewage and other terrestrial environments. A large number of DMS degrading isolates of *Hyphomicrobium* and *Thiobacillus* spp. have been described, but whether these represent the dominant active DMS-degrading populations in terrestrial environments remains unknown (reviewed in Schäfer *et al.*, 2010).

DNA stable isotope probing (SIP) allows relating the function of uncultivated microorganisms to their identities through incorporation of stable isotope labels (for example, ^13^C or ^15^N) into their DNA (Radajewski *et al.*, 2000). SIP has been applied with a range of labelled substrates to identify functionally active microorganisms in the environment (Neufeld *et al.*, 2007b). SIP experiments with ^13^C-labelled methanol, methylamine, formaldehyde and formate have been carried out to identify active methylotrophs in Lake Washington sediments (Nercessian *et al.*, 2005; Kalyuzhnaya *et al.*, 2008). These studies revealed the dominance of *Methylophilaceae* in the SIP enrichments. [^13^C_2_]-DMS was used previously to investigate the active marine methylotrophs during a phytoplankton bloom in the English Channel (Neufeld *et al.*, 2008a), suggesting that DMS degrading bacteria in seawater were *Gammaproteobacteria*, related to but distinct from previously isolated DMS-degrading *Methylophaga* strains (Schäfer, 2007).

Previous studies have demonstrated the potential of SIP combined with metagenomics for the analysis of functionally defined, yet uncultivated microorganisms (Dumont *et al.*, 2006; Kalyuzhnaya *et al.*, 2008; Neufeld *et al.*, 2008b). In the current study, we applied a SIP-metagenomics approach to identify aerobic bacterial populations utilising DMS as a carbon and energy source in a soil and a lake sediment and to gain insight into functional aspects of microbial populations degrading DMS.

## Materials and methods

### Sampling

Soil samples were collected from the top 10 cm of Hunts Mill field with a crop of *Brassica oleraceae* (Wellesbourne, Warwickshire, UK) using an ethanol-sterilised trowel on 20 January 2010. Hunts Mill is an organic sandy loam soil with 73% sand, 12% silt and 14% clay (Whitfield, 1974). A field growing *Brassica oleraceae* was chosen for soil sampling since brassicas have a high content of sulphur containing glucosinolates, which may be released to soil *via* the roots and be subject to microbial degradation that releases volatile sulphur compounds (Crespo *et al.*, 2012). Samples were obtained from five discrete locations in a 2.5 × 1.5 m area and a composite sample was derived. Soil was transferred to the laboratory immediately and kept at 4 °C until SIP incubations were set up. Lake sediment was collected on 08 June 2011 from Tocil Lake, a small eutrophic lake on the campus of the University of Warwick (Coventry, UK). Samples were taken from close to the shore to a depth of 6.5 cm using an acrylic corer and divided into four layers (0–1 cm, 1–2.5 cm, 2.5–4.5 cm and 4.5–6.5 cm) to measure DMS uptake rates. Sediment samples for SIP analysis were collected on 28 June 2011 as above and kept at 4 °C for 1 day until the surface layer was sliced off to a depth of 2.5 cm and homogenised with a spatula.

### DMS uptake

[^12^C_2_]- or [^13^C_2_]-DMS uptake rates of soil and lake sediment samples were measured before time course SIP experiments. [^12^C_2_]-DMS uptake rates of lake sediment samples were measured in triplicate. Two grams of homogenised samples from each layer were placed in 125-ml crimp-top serum vials, sealed with butyl rubber stoppers and amended with 100 nanomoles of [^12^C_2_]-DMS. A gas chromatograph (Agilent Technologies, Cheshire, UK) fitted with a 30 m × 0.32 mm column (DB-1) and a flame ionisation detector was used to monitor the concentration of DMS in the headspace of the incubation vials. Helium was used as the carrier gas at a temperature of 200 °C. Similarly, an experiment was carried out to assess [^12^C_2_]- and [^13^C_2_]-DMS uptake by the soil samples. Triplicate microcosms were set up using 1 g of soil sample and amended with [^13^C_2_]- or [^12^C_2_]-DMS as described above. ^13^C-labelled DMS was synthesised as described previously (Neufeld *et al.*, 2008a). DNA extraction, fractionation and PCR/denaturing gradient gel electrophoresis (DGGE) analysis were carried out as described below and in Supplementary Information to confirm that DMS uptake and label incorporation occurred.

### Stable isotope probing

Stable isotope probing (SIP) of DMS-degrading bacteria was carried out according to Neufeld *et al.* (2007b). Three sets of triplicate microcosms were prepared using 1 g of wet soil or lake sediment sample per microcosm and amended with 450 nmoles per gram of DMS initially. GC measurements were taken twice a day and more DMS was provided to the incubation bottles when it was depleted. [^12^C_2_]-DMS incubations were set up as controls to assess potential differences in the rate of DMS oxidation by microorganisms during SIP incubations using the heavy isotope-labelled [^13^C_2_]-DMS.

### DNA extraction and gradient fractionation

DNA was extracted from approximately 500 mg of sample from the microcosms using the Fast DNA soil extraction kit (MP Biomedicals, Santa Ana, CA, USA) according to the manufacturer's instructions. DNA concentrations were estimated using a spectrophotometer (NanoDrop ND-1000, Nanodrop products, Wilmington, DE, USA) and found to be in a range of 150–200 ng μl^−1^. In all, 15–20 μl aliquots of DNA (3 μg of DNA) were subjected to ultracentrifugation and DNA was recovered *via* gradient fractionation as described previously (Neufeld *et al.*, 2007c).

### Pyrosequencing of 16S rRNA genes

Pyrosequencing of the hypervariable regions (V1 and V2) of bacterial 16S rRNA genes was performed using a Roche 454 GS Junior (Roche 454 Life Sciences, Branford, CT, USA) at Micropathology Ltd. (Coventry, UK). The manufacturer's Universal Tailed Amplicon Sequencing protocol was applied with primers 27 F (Lane, 1991) and 519 R (Muyzer *et al.*, 1993). Analyses of the pyrosequencing data were performed using a pipeline through the Quantitative Insights Into Microbial Ecology (QIIME) software, version 1.6.0 (Caporaso *et al.*, 2010). Details can be found in Supplementary Material. Data have been submitted to the NCBI read archive under bioproject number PRJNA247775.

### Metagenomics

Multiple displacement amplification was carried out to obtain micrograms of DNA from the ‘heavy' (fraction 7) and ‘light' (fraction 12) DNA of the second time-point SIP incubations. For this purpose, 100 times diluted templates were amplified using the Illustra GenomiPhi V2 DNA amplification kit (GE Life Sciences, Little Chalfont, UK) according to the manufacturer's instructions. PCR-DGGE analysis of phi29 amplified DNA did not reveal major changes in community fingerprints due to multiple displacement amplification (Supplementary Figure 1). Metagenomic libraries of the Genomiphi-amplified ^13^C-labelled DNA from ‘heavy' and ‘light' fractions were sequenced using the Illumina HiSeq2000 technology (GATC Biotech, Konstanz, Germany) which generated 8–12 Gb of 100 bp paired-end sequence data per sample. Read data have been submitted to the EBI short read archive under study accession PRJEB6310.

### Assembly, annotation and analysis of metagenomes

Raw metagenomic reads from all samples were assembled into contigs using Ray Meta, an open source, *de novo* metagenome assembler (Boisvert *et al.*, 2012). Assembled sequences were then annotated using Metagenomics Rapid Annotation using Subsystem Technology (MG-RAST) server version 3.2 (Meyer *et al.*, 2008) and compared against the M5NR and Subsystems databases for taxonomic and functional distributions, respectively. In both cases, the maximum *e*-value cutoff was 1*e*−5, minimum identity cutoff was 60% and minimum alignment length cutoff was 15. The assembled contigs obtained by Ray Meta were also screened for functional genes involved in methylotrophy and sulphur metabolism using the BLAST search programme (Altschul *et al.*, 1990) (see Supplementary Information). Assembled contigs are available on MGRAST (IDs 4528621.3, 4528622.3, 4528623.3 and 4528624.3). Taxonomic profiling based on unassembled metagenomic read data was carried out with Kraken (Wood and Salzberg, 2014). Sample reads were searched against a custom-made MiniKraken database that includes Archaea, Bacteria, Virus, Protozoa, Fungi and Plant RefSeq (NCBI Reference Sequence) data sets. Additional detail of Kraken analysis is given in the Supplementary Information.

### Statistical analysis

Details of statistical analyses of 454 amplicon sequencing and metagenomic assemblies are given in the Supplementary Information. Briefly, principal components analysis (PCA) was applied to determine the operational taxonomical units (OTUs) primarily contributing to the differences in pyrosequencing data sets. This was followed by canonical variates analysis to identify the OTUs contributing to the discrimination of the samples by both time and treatment (^12^C or ^13^C). Analysis of variance and least significant difference (LSD) tests were further applied to the key OTUs identified by PCA to find significant differences at the 5% level. Metagenomics profiles obtained by MG-RAST (Meyer *et al.*, 2008) were analysed using Statistical Analysis of Metagenomic Profiles (STAMP) software version 2.0 (Parks and Beiko, 2010). Two-sided Fisher's exact test was applied to compare the family-level proportions in the ‘heavy' and ‘light' DNA fractions.

## Results

### Assessment of DMS uptake potentials in lake sediment and soil samples

The top two layers of the lake sediment were found to be the most active in terms of [^12^C_2_]-DMS uptake with a rate up to 53.6±0.91 nmol DMS g^−1^ (wet weight) sediment day^−1^. Therefore, SIP incubations were set up using the samples from the top layers (0–2.5 cm) of the lake sediment.

Uptake of [^12^C_2_]- and [^13^C_2_]-DMS into soil was also assessed using a total amount of 15 μmoles of DMS amended to the incubations in 20 days. DGGE fingerprinting of the fractions (Supplementary Figure 2) showed that a restricted bacterial community consumed both ^12^C- and ^13^C- labelled DMS. A distinct pattern was observed in the ‘heavy' part of the [^13^C_2_]-DMS fractions (fractions 7 and 8) when compared with the ‘light' part of the same microcosm (fractions 11 and 12) representing the rest of the bacterial community that did not assimilate the [^13^C_2_]-DMS. Three dominant bands (Supplementary Figure 2, bands 1–3) were common in both ‘heavy' DNA fraction of the [^13^C_2_]-DMS incubation and ‘light' DNA fraction of the [^12^C_2_]-DMS incubation indicating the potential DMS degraders in the soil sample as these bands disappeared on the ‘light' fractions of the [^13^C_2_]-DMS incubation. Two of these bands had high identity to 16S rRNA genes of species from the *Methylophilaceae* family while the third represented a species of *Thiobacillus* (Supplementary Table 1).

### Time course SIP experiments

The soil incubations were terminated after the samples had consumed approximately a total of 2.7, 8 and 15 μmoles of DMS per gram of soil (days 11, 17 and 20, respectively; Supplementary Figure 3a). The lake sediment incubations were terminated after approximately 5, 10 and 20 μmoles of DMS per gram of sediment had been consumed (days 10, 12 and 16, respectively; Supplementary Figure 3b). Initial microbial diversity analysis of DNA from lake sediment SIP gradients was carried out using DGGE (Supplementary Figure 4) and sequencing of dominant bands suggested proliferation of *Methylophilus* and *Thiobacillus* spp. (Supplementary Table 1).

### 454-analysis of bacterial diversity in SIP gradient fractions

**Figure 1:**
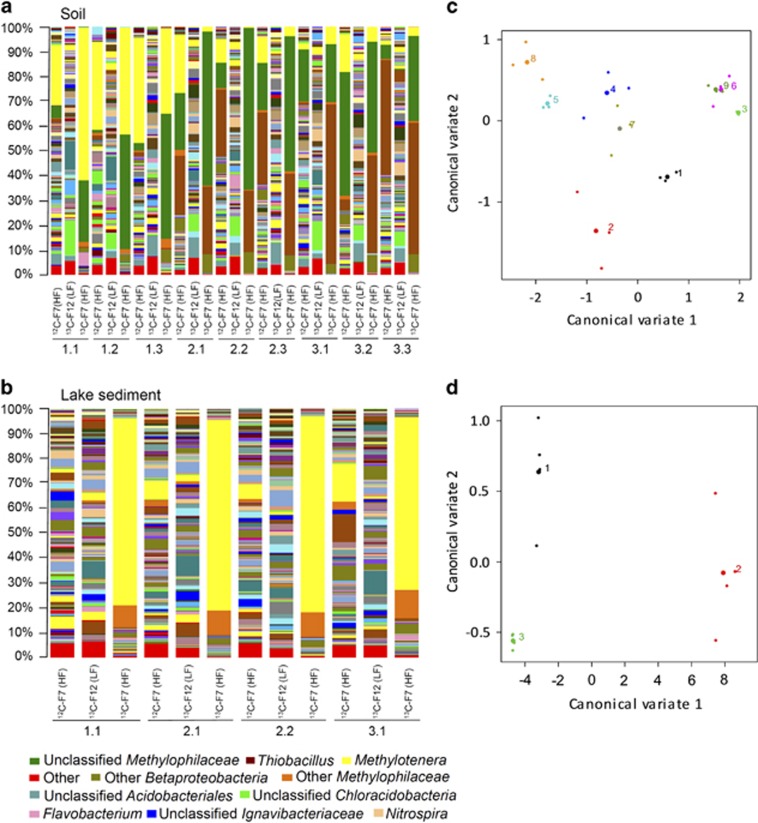
Genus-level taxonomic profiling of the pyrosequencing data sets of 16S rRNA genes of heavy (fraction 7; F7) and light fractions (fraction 12, F12) from [^12^C_2_]-DMS or [^13^C_2_]-DMS incubated microcosms using (**a**) soil sample or (**b**) lake sediment sample. (**c**, **d**) Ordination of canonical variates (CV1 and CV2) produced from canonical variate analysis of pyrosequencing data at family level. Sample labels include the fraction number (F7 (HF) or F12 (LF)) and type of carbon used (^12^C or ^13^C). Numbers under each plot refer to the SIP experiment time point and replicate number. *i.e.* 2.1: second time point, replicate 1. Numbers in (**c**) represent nine different SIP fractions from the soil microcosm samples. (1) Time point 1, ^12^C ‘heavy' fraction (F7), (2) Time point 1, ^13^C ‘heavy' fraction (F7), (3) Time point 1, ^13^C ‘light' fraction (F12), (4) Time point 2, ^12^C ‘heavy' fraction (F7), (5) Time- point 2, ^13^C ‘heavy' fraction (F7), (6) Time point 2, ^13^C ‘light' fraction (F12), (7) Time point 3, ^12^C ‘heavy' fraction (F7), (8) Time point 3, ^13^C ‘heavy' fraction (F7), (9) Time point 3, ^13^C ‘light' fraction (F12). Numbers in (**d**) represent three different treatments in the lake sediment microcosm samples. (1) ^12^C ‘heavy' fraction (F7), (2) ^13^C ‘heavy' fraction (F7), (3) ^13^C ‘light' fraction (F12). Big dots represent the means and small dots represent the individual observations. Note that only the most predominant taxa in each fraction are colour-annotated, including *Methylotenera* (yellow), unclassified *Methylophilaceae* (green), *Thiobacillus* (brown), other *Methylophilaceae* (amber) and other *Betaproteobacteria* (dark green). The relative abundances of the 15 most abundant genera found in the ‘heavy' DNA fractions identified in the pyrosequencing data are shown in the Supplementary Table 2.

The ‘heavy' and ‘light' SIP gradient fractions from the three time points were further analysed by pyrosequencing of the 16S rRNA genes. A total of 31 401 quality-filtered, chimera-free sequences were obtained from 39 samples. These sequences were assigned to 9080 distinct OTUs at 97% identity (average 805±327 reads per sample with an average length of 481 bp). Taxonomic analysis revealed distinct community profiles in the ‘heavy' and ‘light' DNA fractions of the soil and lake sediment SIP samples (Figures 1a and b; Supplementary Table 2). The changes in the read abundance for particular OTUs in different DNA fractions and across the time course of the incubations suggested the degradation and assimilation of ^13^C-labelled DMS by specific bacterial populations. PCA supported the difference between the bacterial communities in DMS-SIP samples (Supplementary Figure 5). The first four principal components explained 45.86% and 59.88% of the total variability in the soil and lake sediment samples, respectively. Canonical variates analysis of the data clearly separated ^13^C-labelled ‘heavy' DNA from ^13^C-labelled ‘light' DNA fractions from different time points (Figures 1c and d). *Betaproteobacteria* dominated the ^13^C-labelled ‘heavy' fractions (F7) of all samples with percentages between 87.5 and 99.5. At the family level, members of the *Methylophilaceae* were prevalent across both soil and lake sediment ^13^C-labelled ‘heavy' DNA samples.

**Figure 2:**
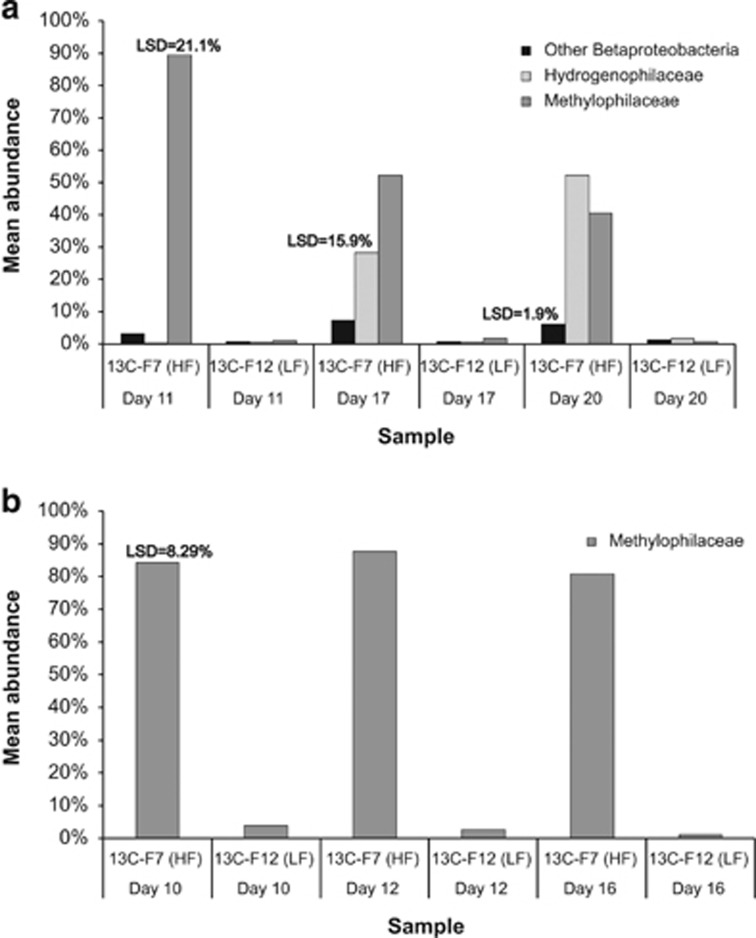
Mean abundances and LSD values of *Methylophilaceae* (dark grey), *Hydrogenophilaceae* (light grey) and other *Betaproteobacteria* (black) in ^13^C-labelled ‘heavy' and ‘light' fractions from each time point. Note that only families which had significantly higher abundances in the ‘heavy' fractions were shown. F7: ‘heavy' fraction F12: ‘light' fraction. (**a**) Soil, (**b**) Lake sediment.

In the soil DMS-SIP, *Methylophilaceae* comprised an average of 89.4% (±1.5%) of the sequences from the ^13^C-labelled ‘heavy' fractions in the first time-point samples (day 11; 2.7 μmoles of DMS), decreasing to 63.2% (±3.7%) and 40.5% (±10.4%) in the second (day 17; 8 μmoles of DMS) and third time-point samples (day 20; 15 μmoles of DMS), respectively. The mean *Methylophilaceae* read abundances in the ^13^C-labelled ‘heavy' fractions were significantly higher than those in the ^13^C-labelled ‘light' fractions at each time point (LSD=21%, *P*=0.028; Figure 1a and Figure 2a). When the data sets were grouped according to the treatment only (^12^C or ^13^C-labelled DMS), the pairwise difference between the *Methylophilaceae* read abundances was even more pronounced (64% and 1% for ^13^C-labelled ‘heavy' and ‘light' fractions, respectively; LSD=12%, *P*=0.00). There was a concomitant increase in the mean read abundance of *Hydrogenophilaceae* in the ^13^C-labelled ‘heavy' fractions from 0.2% in the first time point (day 11) to 28.4% (±4.1%) and 52.2% (±12.4%) in the second (day 17) and third time points (day 20), respectively. Similarly, the mean read abundances of *Hydrogenophilaceae* in the ^13^C-labelled ‘heavy' fractions from the second and third time points were significantly higher than that of the ^13^C-labelled ‘light' fractions (LSD=15.9%, *P*=0.0018; Figures 1a and 2a). *Hydrogenophilaceae* sequence abundances contributed significantly (*P*=0.0001) to the discrimination of the samples by treatment only (26.9% and 1% for ^13^C-labelled ‘heavy' fraction and ^13^C-labelled ‘light' fraction; LSD=9.2%). Other *Betaproteobacteria* also increased in the second and third time point ‘heavy' fractions (7.4±1.1% and 6.1±2.1%, respectively) compared with the first time point (3.2±0.8%). Likewise, there was a significant pairwise difference between ^13^C-labelled ‘heavy' and ‘light' fractions (LSD=1.9%, *P*=0.047; Figure 2a). At the genus level, the *Methylophilaceae* were separated into *Methylotenera* and unclassified *Methylophilaceae* sequences in the first time-point (day 11) soil samples. Their relative abundances decreased while *Thiobacillus* (*Hydrogenophilaceae*) abundance increased in the second and third time-point (days 17 and 20, respectively) samples (Figure 1a; Supplementary Table 2a). The ‘light' fractions of the [^13^C_2_]-DMS microcosms were not enriched with *Methylophilaceae* or *Hydrogenophilaceae* sequences, but comprised highly diverse bacterial communities representing the soil bacterial community. Interestingly, the ‘heavy' fraction of the ^12^C-control microcosm of the soil sample had an increase in read abundance for *Methylophilaceae* and *Thiobacillus* species in addition to the background microbial community, suggesting the proliferation of these organisms on DMS in the ^12^C-microcosm that lead to their dominance in the microbial community.

The lake sediment DMS-SIP ^13^C-labelled ‘heavy' fractions were similarly dominated by members of the *Methylophilaceae*, comprising 84.2% of the sequences from the first time-point sample (day 10; 5 μmoles of DMS). Their read abundance increased to an average of 87.6% in the second time-point replicates (day 12; 10 μmoles of DMS) and decreased to 80.9% in the third time-point sample (day 16; 20 μmoles of DMS). The pairwise differences between the *Methylophilaceae* read abundances in the ^13^C-labelled ‘heavy' and ‘light' fractions at each time point were significant (LSD=8.2%, *P*=0.036; Figures 1b and 2b). This was also the case when the data sets were grouped by treatment only (85% and 2.5% for ^13^C-labelled ‘heavy' and ‘light' fractions, respectively; LSD=4.1%, *P*=0.00). *Methylotenera* remained the most dominant genus in the lake sediment samples from the three time points (Figure 1b; Supplementary Table 2b). While *Thiobacillus* spp. detected by DGGE band sequencing in the lake sediment ‘heavy' DNA were also detected by 454 amplicon sequencing, the read abundance remained fairly constant and they were not found as one of the OTUs contributing to the differences between the data sets according to the PCA which suggests that these did not proliferate significantly. As for the soil DMS-SIP experiment, the ‘light' DNA fractions comprised diverse microbial communities with only minor increase in read abundance of *Methylotenera* related bacteria from the second to third time point (days 12 and 16, respectively).

### Taxonomic profiling of the metagenomes

Average contig lengths were 1279 and 1728 bp for the ‘light' and ‘heavy' fractions of the [^13^C_2_]-DMS soil incubation, respectively (Table 1). Based on all the protein annotation sources (M5NR database) used by MG-RAST, the ‘heavy' and ‘light' metagenomes were dominated by bacteria (74.9 and 76.9%). The proportions of *Archaea* or *Eukarya* were not >6.4% in either fraction while unassigned sequences comprised 13.4% and 14.8% of the ‘heavy' and ‘light' metagenomes, respectively. Kraken analysis showed that of the classified reads in the ‘heavy' fraction of soil SIP, 85.9% were bacterial, 0.17% were archaeal and 0.87% viral DNA, while in the ‘light' fraction of soil SIP 17.2% were bacterial, 0.61% were archaeal and 1.48% were of viral origin. Eukaryotic sequences (fungi, protozoa, plant and algae) comprised 13% and 80.6% of the soil ‘heavy' and ‘light' fractions, respectively.

**Figure 3:**
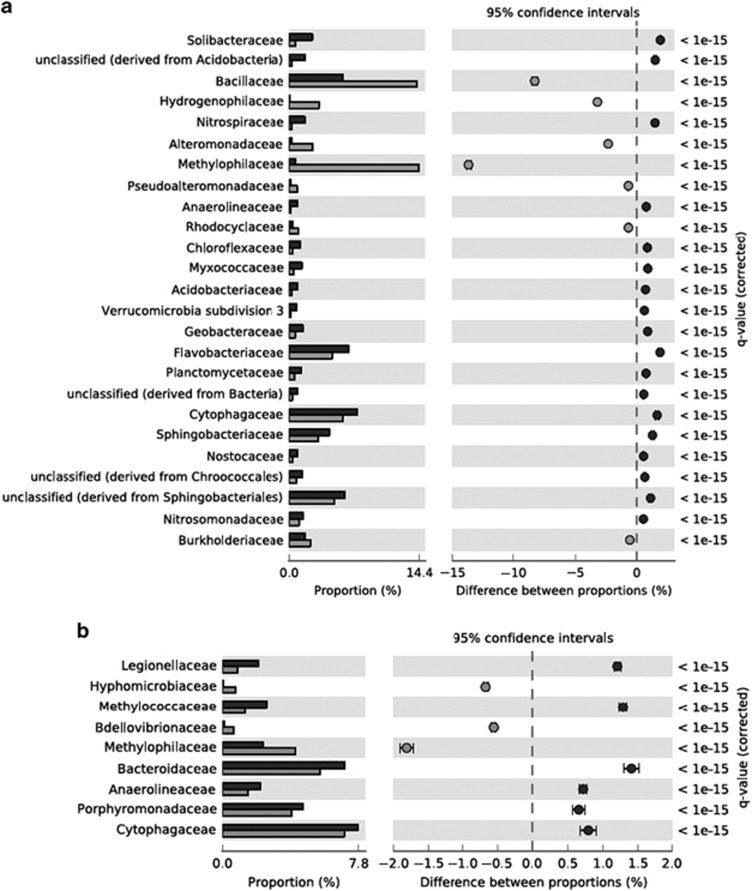
Family-level taxonomic analysis of metagenomic sequences obtained from ‘light' and ‘heavy' DNA samples from the SIP incubations using the STAMP software. Dark grey bars represent the ‘light' DNA and light grey bars represent the ‘heavy' DNA. *Methylophilaceae* family had the greatest proportional difference in the both ‘heavy' metagenomes. (**a**) Soil, (**b**) Lake sediment.

*Proteobacteria* and *Bacteroidetes* predominated the soil ‘heavy' and ‘light' fractions with 45.8% and 32.8%, respectively. *Sphingobacteria* dominated the sequences among the *Bacteroidetes* in the ‘light' fraction (34.7%) while *Betaproteobacteria* dominated in the ‘heavy' fraction (60.9%). With increasing taxonomic resolution at the family level, unclassified *Sphingobacteriales* was the most dominant group in the ‘light' metagenome with 5.0% abundance. However, in the ‘heavy' metagenome, *Methylophilaceae* were the most abundant family (11.1%). According to STAMP comparison, the *Methylophilaceae* family was also indicated as the most overrepresented family in the ‘heavy' fraction (Figure 3a). STAMP comparisons of the ‘heavy' and ‘light' fractions at the genus level revealed that *Methylovorus*, *Methylotenera* and *Methylobacillus* were the most overrepresented genera within the *Methylophilaceae* family in the ‘heavy' metagenome (Supplementary Figure 6a). *Bacillus* was the most abundant genus in the ‘heavy' fraction (11%).

For the lake sediment metagenomes, the average contig lengths were 1230 and 1286 bp for the ‘light' and ‘heavy' fractions, respectively (Table 1). Similar to the soil metagenomes, bacteria dominated the sequences in both fractions with 81.8% and 81.4%. Unassigned sequences, *Archaea* and *Eukarya* comprised 12.8, 2.4 and 2.4% of the ‘heavy' metagenome, and 12.8%, 3.9% and 1.0%, in the ‘light' metagenome, respectively. Kraken analysis showed that of the classified reads in the ‘heavy' fraction of the lake sediment SIP, 71.7% were bacterial, 1.68% were archaeal and 3.3% viral DNA, while in the ‘light' fraction of lake sediment SIP 46.13% were bacterial, 6.6% were archaeal and 4.2% were of viral origin. In all, 23.2% and 43% were Eukaryotic sequences (fungi, protozoa, plant and algae) in the ‘heavy' and ‘light' fractions, respectively.

Taxonomic assignments of the lake metagenomic sequences showed differences compared with the soil metagenome. In both fractions, *Bacteroidetes* was the most dominant phylum (37.8% and 38.4% of the ‘light' and ‘heavy' fractions, respectively) followed by *Proteobacteria* which increased from 24.5% in the ‘light' fraction to 38.4% in the ‘heavy' fraction. Within *Proteobacteria*, the most abundant class in the ‘light' fraction was *Gammaproteobacteria* (39.8%) whereas *Betaproteobacteria* was the most abundant in the ‘heavy' fraction with 30.3%.

Comparison of the ‘heavy' and ‘light' fractions from the lake sediment SIP incubations by STAMP software indicated *Methylophilaceae, Hyphomicrobiaceae* and *Bdellovibrionaceae* as the most overrepresented taxa (Figure 3b). At the genus level, the fractional abundances of *Methylobacillus*, *Methylotenera, Hyphomicrobium* and *Bdellovibrio* sequences significantly increased in the ‘heavy' fraction (Supplementary Figure 6b).

Analysis of unassembled reads using Kraken also suggested that *Methylophilaceae* and *Hydrogenophilaceae* increased in abundance in the soil ‘heavy' SIP fractions, although the estimate of the fractional abundances (15.4% and 17.1%, respectively) was lower than that derived by amplicon sequencing. Similarly, Kraken analysis estimated the *Methylophilaceae* fractional abundance to be 45.5% and no marked increase of *Hydrogenophilaceae* in the lake sediment ‘heavy' fraction (Supplementary Figure 7). Overall, Kraken analysis had low fraction of matching kmers in the unassembled read data sets ranging between 1.4% and 6.9% suggesting that the coverage of the uncultivated microorganisms in the database was poor (Supplementary Table 3).

### Functional profiling of the metagenomes

**Figure 4:**
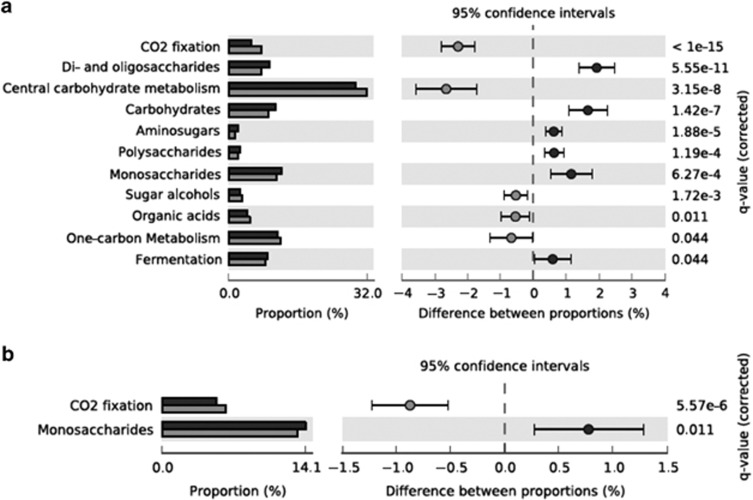
Functional analysis of metagenomic sequences from the carbohydrate metabolism category (MG-RAST) obtained from ‘light' and ‘heavy' DNA samples from the SIP incubations using the STAMP software. Dark grey bars represent the ‘light' DNA and light grey bars represent the ‘heavy' DNA. (**a**) Soil, (**b**) Lake sediment.

Analysis of the MG-RAST subsystems category ‘carbohydrate metabolism' in soil fractions revealed the over-representation of ‘central carbohydrate metabolism', ‘CO_2_ fixation', ‘one-carbon metabolism', ‘sugar alcohols' and ‘organic acids' in the ‘heavy' metagenome (Figure 4a). Within the category ‘one-carbon metabolism', the percentage of the sequences from the serine-glyoxylate cycle decreased from 91.9% in the ‘light' to 87.3% in the ‘heavy' fractions (corrected *P*-value=1.24*e*−7). However, the abundance of the sequences representing the ribulose-monophosphate pathway significantly increased from 0.30% to 1.3% in the ‘heavy' fraction (corrected *P-*value=1.09*e*−5; Supplementary Figure 8a). Sequences matching to ‘CO_2_ fixation' genes occurred significantly more in the ‘heavy' metagenome than in the ‘light' metagenome (corrected *P-*value=<1*e*−15). Among the CO_2_ fixation genes, sequences from the ‘Calvin-Benson cycle' were the most dominant in both fractions (36.8% and 43.9%). However, the number of genes for ‘CO_2_ uptake' and ‘carboxysome' increased significantly in the ‘heavy' fraction (corrected *P-*value=7.56*e*−10 and 3.25*e*−4, respectively). The abundance of genes related to sulphur metabolism was also investigated (Supplementary Figure 9a). In comparison with the ^12^C-DNA fraction, the most overrepresented genes in the ‘heavy' metagenome were involved in ‘inorganic sulphur assimilation', ‘sulphur oxidation' and ‘sulphate reduction-associated complexes' (corrected *P-*value=2.70*e*−5, 3.29*e*−10 and <1*e*−15, respectively).

For the lake sediment metagenomes, within MG-RAST subsystems category ‘carbohydrate metabolism', only genes related to ‘CO_2_ fixation' were significantly more abundant in the ‘heavy' metagenome (corrected *P*-value=5.57*e*−6; Figure 4b). Within the one-carbon metabolism, only the number of sequences related to the ‘serine-glyoxylate cycle' was found significantly more abundant according to the STAMP analysis (corrected *P-*value=1.07*e*−3, Supplementary Figure 8b). The abundance of ‘sulphur oxidation' genes within ‘sulphur metabolism' category was significantly higher in the ‘heavy' fraction (corrected *P*-value=2.77*e*−4; Supplementary Figure 9b).

**Figure 5:**
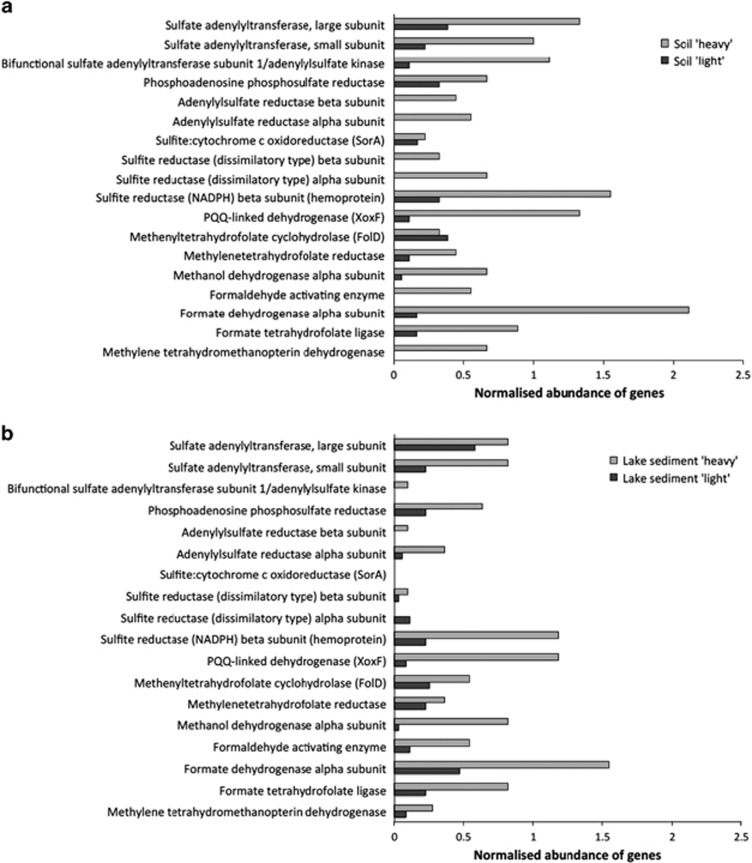
Relative abundance of methylotrophy- and sulphur-related functional genes relative to *recA* (single copy control gene) in ‘heavy' and ‘light' metagenomes. Dark grey bars represent the ‘light' DNA and light grey bars represent the ‘heavy' DNA. (**a**) Soil, (**b**) Lake sediment. Numbers are based on features identified in assembled reads of the metagenomes as shown in Supplementary Table 4.

Additionally, genes encoding for enzymes involved in methylotrophy and oxidation of sulphide and sulphite were searched within the metagenomes using BLAST to identify the affiliation of the relevant genes and to compare their relative abundance to the *recA* gene, which is known to exist as a single copy in bacterial genomes (Eisen, 1995; Wu *et al.*, 2011) (Supplementary Information). A higher relative abundance of genes encoding enzymes of tetrahydrofolate and tetrahydromethanopterin linked C1 oxidation, formaldehyde activating enzyme, formate oxidation as well as methanol dehydrogenase and PQQ linked dehydrogenase was found in ‘heavy' metagenomes (Figure 5a). The gene encoding the large subunit of dimethylsulphide monooxygenase (*dmoA*) was not found in metagenomes. Similarly, the relative abundance per *recA* copy of several genes encoding enzymes involved in inorganic sulphur compound oxidation/assimilation was higher in the ‘heavy' fraction, including flavocytochrome *c* sulphide dehydrogenase, dissimilatory sulphite reductase, adenylylsulphate reductase and sulphate adenylyltransferase. Similar changes in gene frequency were detected in the metagenome of the ‘heavy' fraction of the lake sediment microcosm (Figure 5b).

The methylotrophy and sulphur compound oxidation genes in the ‘heavy' metagenomes were mainly related to *Methylophilaceae* and *Thiobacillus* species, while those detected in the ‘light' fractions were affiliated with more diverse bacteria including members of the *Bacteroidetes* group that was abundant based on 454 read data. Similarly, the *recA* genes (*e*-value <1*e*−30) in the ‘heavy' soil metagenome were exclusively affiliated with *Methylophilaceae* and *Thiobacillus*, but originated from a wide diversity of bacteria in the ‘light' soil metagenome. In the lake sediment ‘heavy' metagenome, *recA* sequences were mainly originating from *Methylotenera*, *Bdellovibrio* and other bacteria while the diversity of *recA* in the ‘light' fraction reflected a more diverse community (Supplementary Table 4).

## Discussion

The fate of DMS in terrestrial ecosystems and the microbial populations involved in its degradation have remained poorly characterised due to a lack of adequate cultivation-independent tools to study active DMS degrading bacteria. While microbial communities from complex ecosystems such as soils are now routinely analysed using high-throughput sequencing (Tringe *et al.*, 2005; Kurokawa *et al.*, 2007; Sul *et al.*, 2009; Delmont *et al.*, 2012), the polyphyletic nature of DMS degrading microbial taxa means that populations degrading DMS cannot be identified using ribosomal sequence analysis alone.

The analysis of DMS-SIP experiments suggested that DMS degradation in microcosms induced the proliferation of a limited number of populations in the community. DGGE analysis, 454 amplicon sequencing of gradient fractions and taxonomic profiling of metagenomic reads were in agreement, suggesting that the DMS-assimilating bacterial populations were dominated by *Betaproteobacteria*. 454-pyrosequencing data analysis revealed the dominance of species from the *Methylophilaceae* family in all ^13^C-labelled ‘heavy' DNA. While in soil DMS-SIP microcosms, *Thiobacillus* species also had increasing read abundance during the incubation, their lower read abundance in lake sediment SIP microcosms suggested that they were potentially less important in DMS assimilation in the lake sediment samples compared with soils. Metagenomic analysis also suggested that *Bacillaceae* were abundant in both ‘light' and ‘heavy' soil metagenomes, however, this was not supported by 454 amplicon sequencing or analysis of functional genes. While the analyses of the metagenomes showed the dominance of *Methylobacillus* and *Methylotenera* in both ^13^C-labelled ‘heavy' DNA fractions from soil and lake sediment, there were also differences between the experiments with soil and lake sediment, consisting of higher representation of *Methylovorus* and *Thiobacillus* in soil SIP microcosms, while analysis of the lake sediment ‘heavy' metagenome also indicated increased abundance of *Hyphomicrobium* and *Bdellovibrionaceae*. There were differences between the relative abundances of key microbial taxa estimated based on amplicon pyrosequencing, assembled metagenomic data and unassembled read data which are likely reflecting the intrinsic differences of these approaches. While ribosomal RNA gene databases would arguably be the most robust tool to match sequences of uncultivated organisms to taxonomic identifiers, kmer-based approaches, such as in Kraken require 100% matches of kmers and are based on databases that are less representative of uncultivated organisms than is possible with matching of amplicon sequences against rRNA databases. The latter is reflected in the low matching of kmers of raw read data to the Kraken database that only classified between 1.3% and 6.9% of reads in all samples (see Supplementary Information). In addition, estimates based on assembled features against the M5NR database would only assess a subset of sequence information (only the assembled part) while also potentially underestimating abundant species due to the fact that different coverage of such contigs would not be considered further. Despite these differences, each of these approaches agreed that *Methylophilacaea* and *Thiobacillus* were the most important taxa that were markedly enriched in the ‘heavy' fraction of the soil SIP, and that *Methylophilaceae* but not *Thiobacillus* were markedly enriched in the ‘heavy' fraction of the lake sediment SIP.

A critical issue in SIP is cross-feeding, which may give false positive identification of substrate assimilating taxa (Neufeld *et al.*, 2007c). The higher abundance of *Bdellovibrionaceae* in the ‘heavy' metagenomes of the lake microcosms is most likely a consequence of these bacteria predating on the bacteria growing in response to DMS addition rather than an active involvement in methylotrophy or sulphur oxidation. Previous SIP studies have repeatedly shown *Bdellovibrio*-like organisms in experiments with different ^13^C-labelled carbon sources (Morris *et al.*, 2002; Hutchens *et al.*, 2004) and *Bdellovibrio*-like organisms were also successfully investigated using SIP with ^13^C-labelled biomass (Chauhan *et al.*, 2009). Conversely, based on prior knowledge of DMS metabolism from cultivation-dependent studies, it might be argued that the true DMS degraders in the microcosms were *Thiobacillus* species, as a number of *Thiobacillus* spp. have previously been shown to degrade DMS (Sivelä and Sundman, 1975; Smith and Kelly, 1988; Visscher and Taylor, 1993; de Zwart *et al.*, 1997), and that *Methylophilaceae* were labelled in SIP due to assimilation of intermediates of DMS degradation (for example, formate, formaldehyde and methanethiol) released by *Thiobacillus* spp. High abundance of *Methylophilaceae* representatives might be due to the ability of some strains to use formaldehyde as a carbon source (Nercessian *et al.*, 2005). Microcosm experiments carried out with Lake Washington sediment to study the response of the microbial community to methane addition have shown increases of non-methanotrophic *Methylophilaceae* populations alongside those of *bona fide* methanotrophs, showing cooperation between *Methylococcaceae* and *Methylophilaceae* in methane utilisation (Beck *et al.*, 2013), but the abundance of the primary methane degrader (*Methylobacter*) was substantial compared with that of the cooperating *Methylophilaceae* populations (Beck *et al.*, 2013; Oshkin *et al.*, 2014). According to the labelling pattern observed by the 454-pyrosequencing data in our study, the scenario that *Methylophilaceae* were getting labelled *via* cross-feeding is unlikely since *Thiobacillus* spp., the known DMS degrader, were not detected in the first time point of the soil DMS-SIP samples and only became a more dominant part of the community after *Methylophilaceae* were already established. The proliferation of *Thiobacillus* species in soil SIP microcosms could have been due to utilisation of intermediates produced by *Methylophilaceae*, for example, by utilising sulphide/sulphite as energy sources and assimilating ^13^CO_2_, but it is equally possible that *Thiobacillus* populations, which assimilate DMS after its initial oxidation to CO_2_ (Kanagawa and Kelly, 1986), were degrading DMS, which might also explain the increase of the CO_2_ fixation genes in the ‘heavy' metagenomes.

The family *Methylophilaceae* includes obligate and facultative methylotrophs but none of the members of this family has been shown to degrade DMS. We tested several cultures from the *Methylophilaceae* for their ability to degrade DMS including *Methylophilus leisingeri*, *Methylophilus methylotrophus* and *Methylotenera versatilis*, but none of the strains tested was able to degrade DMS, suggesting the available cultivated strains may not be representative of the SIP-detected populations. Comparative genomic analysis of *Methylotenera mobilis* JLW8, *Methylotenera versatilis* 301, *Methylovorus glucosetrophus* SIP3-4 with two other *Methylophilaceae* species (*Methylobacillus flagellatus* KT and unclassified *Methylophilales* strain HTCC2181) suggested that these methylotrophic *Betaproteobacteria* are metabolically versatile (Lapidus *et al.*, 2011) and that closely related but metabolically distinct *Methylophilaceae* strains may co-exist in the same environment (Chistoserdova, 2011a); our data strongly support that suggestion and further indicate that certain *Methylophilaceae* are able to degrade DMS. An increase in the abundance of the genes from the Ribulose-monophosphate pathway in the soil ‘heavy' fraction was in agreement with these bacteria being known to use the Ribulose-monophosphate pathway for formaldehyde assimilation (Chistoserdova, 2011b). The absence of homologues of the DMS monooxygenase enzyme characterised in *Hyphomicrobium sulfonivorans* S1 (Boden *et al.*, 2011) suggests that the dominant DMS assimilating microorganisms detected here use an alternative enzyme for DMS degradation, possibly *via* methanethiol, utilising as yet unidentified methyltransferases that have been suggested to be involved in DMS oxidation in certain *Thiobacillus* isolates (Visscher and Taylor, 1993) and in *Methylophaga thiooxydans* (Boden *et al.*, 2011).

The frequency of genes of methylotrophic metabolism markedly increased in ‘heavy' soil and lake sediment metagenomes and their taxonomic classification supported that these uncultivated *Methylophilaceae* and *Thiobacillus* populations were involved in methylotrophy and oxidation of reduced inorganic sulphur compounds. DMS monooxygenase is unlikely to be the primary enzyme of DMS oxidation in these populations, and thus further studies of the biochemistry of DMS degradation in these groups of bacteria are essential. The methylotrophic populations detected in the ‘heavy' fractions also had genes encoding methanol dehydrogenase genes and XoxF, which was previously shown to be expressed during growth of *Methylophaga thiooxydans* on DMS (Schäfer, 2007). Methylotrophy genes detected in the ‘heavy' metagenomes suggest that these bacteria have the potential to degrade methyl groups of DMS *via* tetrahydrofolate or tetrahydromethanopterin bound intermediates. In the *Thiobacillus* spp. detected, sulphide and sulphite oxidation could occur *via* the dissimilatory sulphite reductase, adenylylsulphate reductase and sulphate adenylyltransferase, but it is less clear which enzymes might be involved in sulphite oxidation in *Methylophilaceae*.

The combination of SIP, 454 sequencing of gradient fractions and metagenomic sequencing proved useful for identifying microbial populations actively involved in DMS cycling in two terrestrial environments. It detected known DMS degraders (*Thiobacillus*) but also implicated a group of methylotrophs not previously realised as DMS degraders, uncultured *Methylophilaceae.* These findings reemphasise the significance of members of the *Methylophilaceae* as widely distributed and versatile methylotrophs (Lapidus *et al.*, 2011; Chistoserdova, 2011a), which have important roles in not only carbon and nitrogen, but also sulphur cycling in terrestrial environments likely using as yet unidentified enzymes to degrade DMS.

## Figures and Tables

**Table 1:** Assembly results of metagenomic reads from the soil and lake sediment SIP fractions

	*Soil*		*Lake Sediment*	
	*Light*	*Heavy*	*Light*	*Heavy*
Total number of contigs	80 987	28 382	53 331	22 094
Total contig length (bp)	103 612 778	49 050 429	65 621 201	28 430 434
Average contig length (bp)	1279	1728	1230	1286
N50 (bp)	1567	2 708	1449	1628
Median (bp)	769	934	767	753

Raw sequences obtained by Illumina sequencing were assembled into contigs using Ray Meta (Boisvert *et al.*, 2012).
