# Supplementary material for: SIP metagenomics identifies uncultivated Methylophilaceae as dimethylsulphide degrading bacteria in soil and lake sediment
Source: ISME J. 2015 Mar 27;9(11):2336–48. doi: 10.1038/ismej.2015.37 (PMC4611497; doi:10.1038/ismej.2015.37)
Supplement: Supplementary Material [file ismej201537x1.doc]

**SIP-metagenomics identifies uncultivated *Methylophilaceae* as dimethylsulfide degrading bacteria in soil and lake sediment**

Özge Eyice^1^, Motonobu Namura^2^, Yin Chen^1^, Andrew Mead^1^*, Siva Samavedam^1^, Hendrik Schäfer^1**^

^1^ School of Life Sciences, University of Warwick, Coventry, CV4 7AL, UK

^2^ MOAC Doctoral Training Centre, University of Warwick, Coventry, CV4 7AL, UK

^*^Current address: Rothamsted Research, Harpenden, Hertfordshire, AL5 2JQ, UK

^**^Correspondence: E-mail: H.Schaefer@warwick.ac.uk

**Supplementary Material**

*File content*

This file contains additional information about molecular biological methods, statistical analyses used with pyrosequencing data and metagenome analysis, and supplementary figures and tables.

*Polymerase chain reaction (PCR) and denaturing gradient gel electrophoresis (DGGE)*

PCR was performed for DGGE analysis of 16S rRNA genes from each fraction using the primers 341F-GC and 907R (Muyzer *et al*., 1998). Amplification conditions were as follows: initial denaturation at 95°C for 5 min, 35 cycles of 95°C for 1min, 55°C for 1 min, 72°C for 1.5 min, a final elongation step at 72°C for 5 min. DGGE was carried out using a Bio-Rad DCode system (Bio-rad, Hercules, CA, USA) with 6% (w/v) polyacrylamide (37.5:1 acrylamide: bisacrylamide) gels containing 30-70% linear denaturant gradient (Schäfer and Muyzer, 2001). Specific DGGE bands were cut from the gel, incubated in 10 µl distilled water overnight and 1 µl of the solution was used as a template for PCR amplification using the same primer set. The purity of the PCR products was confirmed by DGGE analysis and then sequenced with primers 341F and 907R using ABI3100 sequence detection system with BigDye Terminator v3.1 cycle sequencing kit (Applied Biosystems, UK). The sequences were assembled using SeqMan, DNA Star Lasergene 2.0 and analysed using RDP-II Classifier (Wang *et al*., 2007).

*Pyrosequencing data analysis*

Quality filtering and denoising of the pyrosequencing data was carried out using Acacia software, version 1.52-b0 (Bragg *et al*., 2012). Downstream analyses were performed using a pipeline through the Quantitative Insights Into Microbial Ecology (QIIME) software, version 1.6.0 (Caporaso *et al*., 2010a). Operational taxonomic unit (OTU) picking was performed against the Greengenes database (DeSantis *et al*., 2006) at ≥97% identity (Edgar, 2010). Alignment of the sequences to the Greengenes Core reference set was achieved using PyNAST (Caporaso *et al*., 2010b). Chimeras were detected using ChimeraSlayer (Haas *et al*., 2011) and removed from further analyses. Taxonomy assignment was done against the Greengenes reference database (McDonald *et al*., 2012) using RDP Classifier 2.2 for classification (Wang *et al*., 2007).

*Taxonomic profiling of metagenomes based on illumina metagenome sequencing read data*

Kraken, an ultrafast metagenomic sequence classification tool (Wood & Salzberg 2014) was used to assign taxonomic labels to our DNA short reads. A MiniKraken database (~80 GB in size) containing Archaea, Bacteria, Virus, Protozoa, Fungi and Plant sequences which were downloaded from RefSeq (NCBI Reference Sequence) database was built to enable taxonomic profiling. Reads from each of our sample were searched against this custom-made MiniKraken database. This resulted in classification of reads at different taxonomic levels. The relative abundance of taxa in each sample was calculated by defining the total number of classified reads as 100%.

*Screening of assembled metagenomic data for genes of methylotrophic and sulfur metabolism*

The assembled contigs obtained by Ray Meta were also screened for functional genes involved in methylotrophy and sulfur metabolism using the BLAST search program (Altschul *et al*., 1990). The matches were filtered according to the e-value and the ones having e-values below 1e-30 were kept. BLASTP search on The National Center for Biotechnology Information (NCBI) was applied to find out the taxonomic identification of the resulting contigs.

The accession numbers to records on uniprot ([www.uniprot.org](http://www.uniprot.org/)) or databases held at the National Center for Biotechnology Information ([www.ncbi.nlm.nih.gov](http://www.ncbi.nlm.nih.gov/)) of the proteins used to query the assembled contigs by blast searches are as follows: B7SBF8_METME DMOA_HYPSL Dimethyl-sulfide monooxygenase *Hyphomicrobium* *sulfonivorans*; Methanol dehydrogenase alpha subunit, *Methylophilus methylotrophus*; F5RHT9_9RHOO PQQ-linked dehydrogenase XoxF1, *Methyloversatilis universalis* FAM5; C6XB75_METSD Formaldehyde-activating enzyme *Methylovorus* sp. (strain SIP3-4); MTDA_METEA Bifunctional protein MdtA *Methylobacterium* *extorquens* (strain ATCC 14718 / DSM 1338 / AM1); FOLD_METFK Bifunctional protein FolD, *Methylobacillus flagellatus* (strain KT / ATCC 51484 / DSM 6875); B2ZZ98_METME Methylenetetrahydrofolate reductase *Methylophilus methylotrophus*; FRMA_ECOHS S-(hydroxymethyl)glutathione dehydrogenase, *Escherichia coli*; SFGH1_ECOHS S-formylglutathione hydrolase FrmB, *Escherichia coli*; RBL1_THIDA Ribulose bisphosphate carboxylase large chain, *Thiobacillus denitrificans*; Q6QUH8_METME Methylene tetrahydromethanopterin dehydrogenase, *Methylophilus methylotrophus*; C6X9A4_METSD Formate--tetrahydrofolate ligase, *Methylovorus* sp. (strain SIP3-4); C6WYL6_METML Formate dehydrogenase, alpha subunit, *Methylotenera mobilis* (strain JLW8 / ATCC BAA-1282 / DSM 17540); C6WYL4_METML Formate dehydrogenase delta subunit, *Methylotenera mobilis* (strain JLW8 / ATCC BAA-1282 / DSM 17540); C6WYL4_METML Formate dehydrogenase delta subunit, *Methylotenera mobilis* (strain JLW8 / ATCC BAA-1282 / DSM 17540); ADI29585 sulfate adenylyltransferase, small subunit, *Methylotenera* *versatilis* 301; ADI29584 sulfate adenylyltransferase, large subunit, *Methylotenera* *versatilis* 301; YP_003048440 phosphoadenosine phosphosulfate reductase, *Methylotenera mobilis* JLW8; B059DRAFT_02521 Sulfite reductase, beta subunit (hemoprotein), *Thiobacillus denitrificans* DSM 12475; Meth11DRAFT_1072 Sulfite reductase, beta subunit (hemoprotein) *Methylophilaceae* sp. #110, AAZ98438 sulfite reductase; CYSD_ALLVD Cytochrome subunit of sulfide dehydrogenase *Allochromatium vinosum*; DHSU_ALLVD Sulfide dehydrogenase [flavocytochrome c] flavoprotein chain, *Allochromatium vinosum*; P72177_PARDE SoxB protein, *Paracoccus denitrificans*; A1B9M5_PARDP Sulfur dehydrogenase subunit SoxC, *Paracoccus denitrificans*; Q9LCU8_PARDE SoxZ protein, *Paracoccus denitrificans*; Q8KQ39_THIDE Sulfide:quinone oxidoreductase, *Thiobacillus denitrificans*; dissimilatory-type alpha subunit, *Thiobacillus denitrificans* ATCC 25259; AAZ98437 sulfite reductase, dissimilatory-type beta subunit, *Thiobacillus* *denitrificans* ATCC 25259; AF154565_2 sulfite:cytochrome c oxidoreductase subunit A SorA, *Starkeya novella* DSM 506; AF154565_3 sulfite:cytochrome c oxidoreductase subunit B SorB, *Starkeya novella* DSM 506; AAZ98235 adenylylsulfate reductase, alpha subunit, *Thiobacillus denitrificans* ATCC 25259; AAZ98236 adenylylsulfate reductase, beta subunit, *Thiobacillus denitrificans* ATCC 25259; YP_313968 bifunctional sulfate adenylyltransferase subunit 1/adenylylsulfate kinase, *Thiobacillus denitrificans* ATCC 25259; RECA_ECOLI Protein RecA *Escherichia coli* (strain K12).

*Statistical analysis*

Principal Components Analysis (PCA) was applied to the correlation matrices for the relative abundance of family level OTUs determined by 454 sequence analysis to identify the OTUs primarily contributing to the differences in pyrosequencing datasets between samples. Canonical Variates Analysis (CVA) was further applied to the same datasets to identify OTUs primarily contributing to the discrimination of the samples by both time points and treatment (^12^C or ^13^C). For key OTUs identified from the PCAs (with magnitude of the coefficients greater than 0.1 in the first four principal components), analysis of variance (ANOVA) was applied to identify where there was evidence of differences in relative abundance between time points, treatments, and for combinations of time point and treatment. Where terms were identified to be significant (above 5% level), least significant differences (LSDs) (5%) were used to find the particular differences that were significant. All analyses were performed using GenStat v15 (VSN International Ltd., Hemel Hempstead, UK).

Metagenomic profiles obtained by MG-RAST (Meyer *et al*., 2008) were analysed using the Statistical Analysis of Metagenomic Profiles (STAMP) software version 2.0 (Parks and Beiko, 2010). Two-sided Fisher’s exact test was applied to the metagenomes to compare the proportions of families in the ‘heavy’ and ‘light’ DNA (Agresti, 1990). Benjamini-Hochberg False Discovery Rate (FDR) method was then used to control the incorrectly rejected null hypotheses and adjust the significance level for multiple comparison (Benjamini and Hochberg, 1995). The confidence interval was also adjusted for multiple comparison with continuity correction using DP: asymptotic-CC confidence interval method (95% confidence level; Newcombe, 1998). Results were filtered using the p-value filter (>0.05) and the effect size filter (difference between proportions <0.50%).

**Supplementary Figures**

**
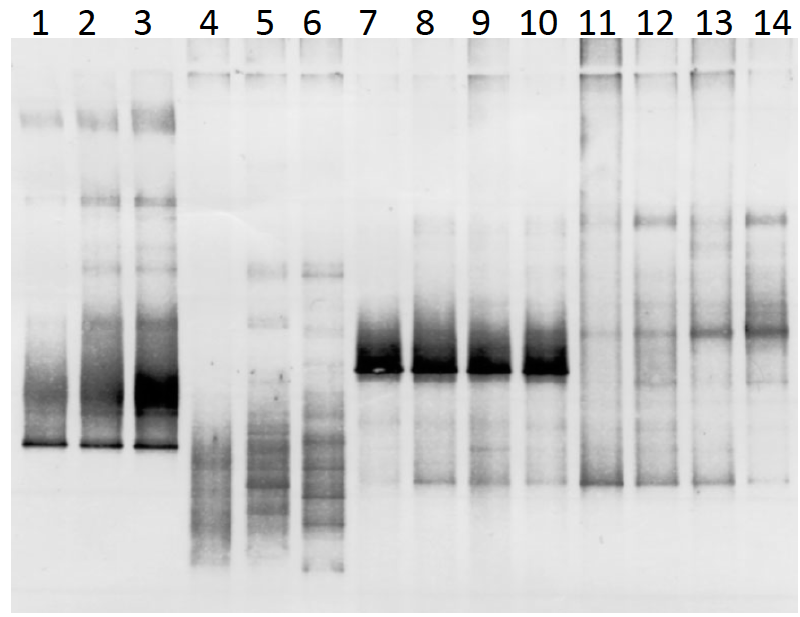
**

**Supplementary Figure 1**. DGGE gel image showing the 16S rRNA gene PCR products of original and phi29 amplified ‘heavy’ and ‘light’ DNA fractions from the soil and lake sediment SIP experiments. Lane 1: Soil ‘heavy’ fraction (F7) original DNA, 2: 1/100 diluted and phi29 amplified DNA from the soil ‘heavy’ fraction (F7), 3: Replicate of 1/100 diluted and phi29 amplified DNA from the soil ‘heavy’ fraction (F7), 4: Soil ‘light’ fraction (F12) original DNA, 5: 1/100 diluted and phi29 amplified DNA from the soil ‘light’ fraction (F12), 6: Replicate of 1/100 diluted and phi29 amplified DNA from the soil ‘light’ fraction (F12), 7: Lake sediment ‘heavy’ fraction (F7) original DNA, 8: 1/100 diluted and phi29 amplified DNA from the lake sediment ‘heavy’ fraction (F7), 9: Lake sediment ‘heavy’ fraction (F8) original DNA, 10: 1/100 diluted and phi29 amplified DNA from the lake sediment ‘heavy’ fraction (F8), 11: Lake sediment ‘light’ fraction (F12) original DNA, 12: 1/100 diluted and phi29 amplified DNA from the lake sediment ‘light’ fraction (F12), 13: Lake sediment ‘light’ fraction (F13) original DNA, 14: 1/100 diluted and phi29 amplified DNA from the lake sediment ‘light’ fraction (F13).


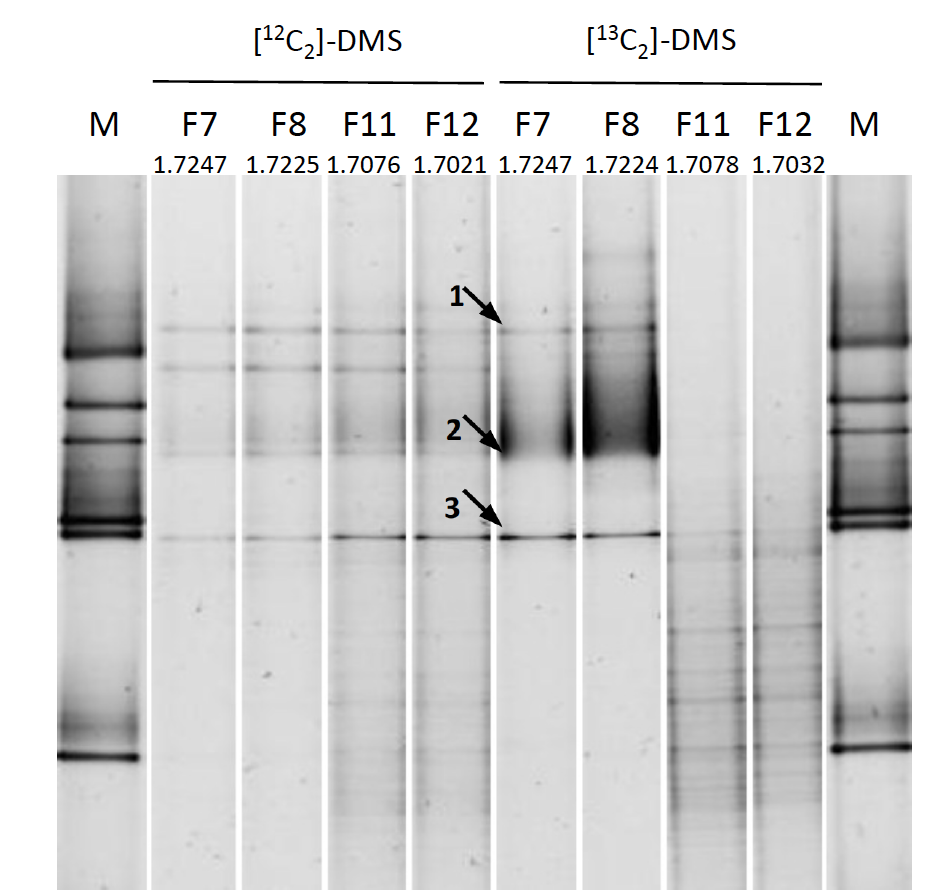


**Supplementary Figure 2**. DGGE gel image showing the bacterial 16S rRNA gene fingerprints of the ^12^C and ^13^C-labelled DNA recovered from the ‘heavy’ and ‘light’ fractions of the soil DMS-uptake incubations after 15 μmoles of DMS was consumed. Fraction numbers and the buoyant densities (g/ml) of each fraction were shown above each lane on the picture. Sequenced bands are indicated with arrows. M: DGGE marker.

**Supplementary Figure 3**. Estimated DMS amounts degraded in representative SIP microcosms based on the GC measurements during the time-course experiment. Arrows show the days when each set was sacrificed for DNA extraction and further analyses. (a) Soil, (b) Lake sediment.


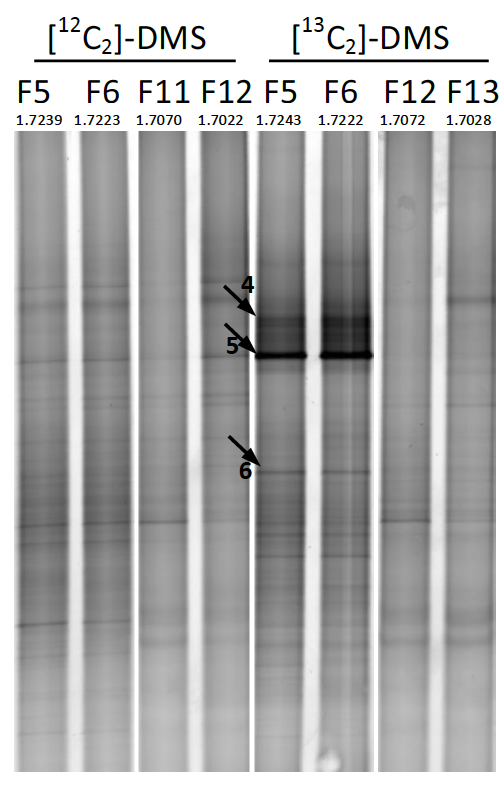


**Supplementary Figure 4**. DGGE gel image showing the bacterial 16S rRNA gene fingerprints of the ^12^C and ^13^C-labelled DNA recovered from the ‘heavy’ and ‘light’ fractions of the second time-point lake sediment SIP incubations after 10 μmoles (per gram sediment) of DMS was consumed. Fraction numbers and the buoyant densities (g/ml) of each fraction were shown above each lane on the picture. Sequenced bands are indicated with arrows.


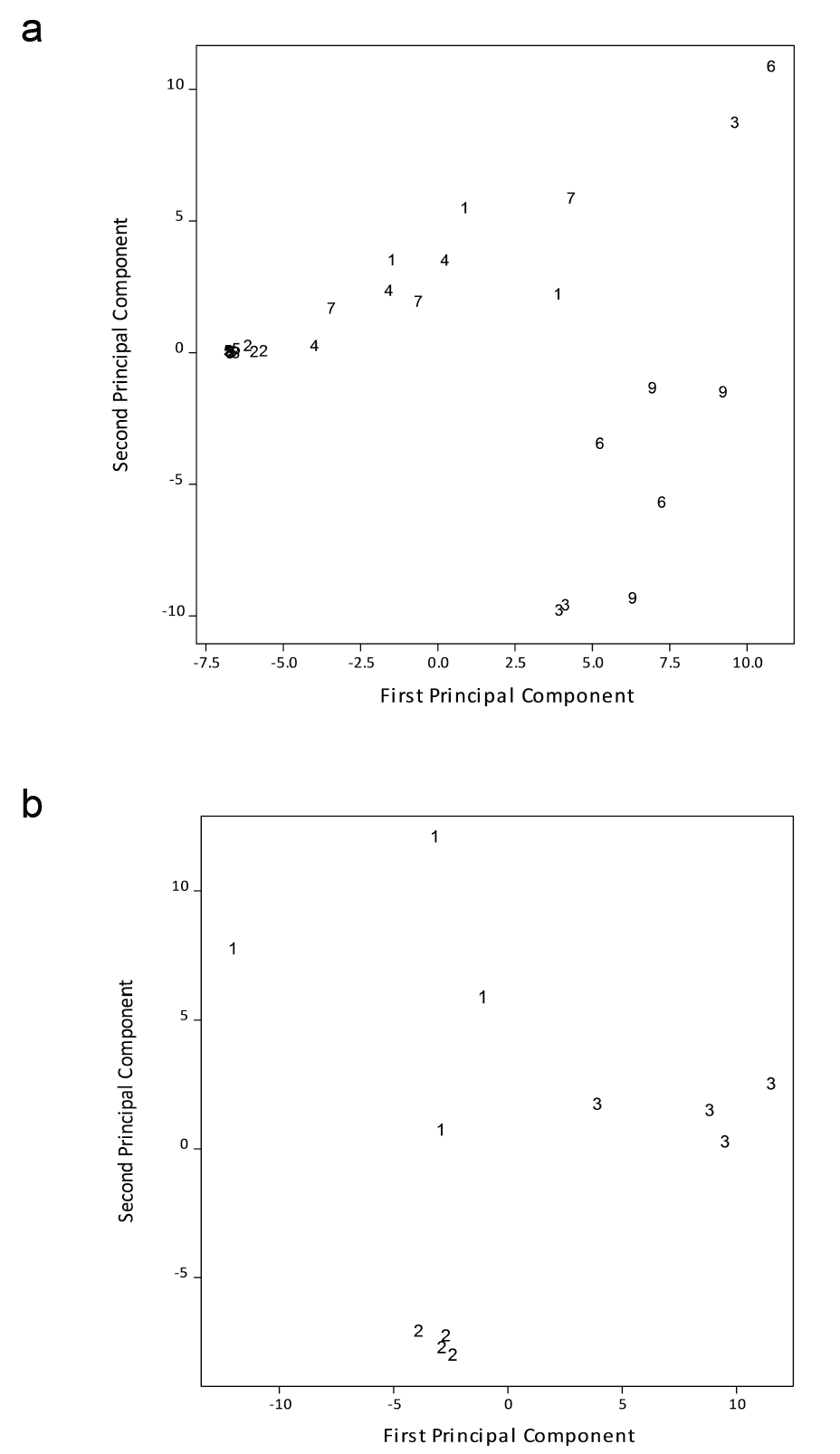


**Supplementary Figure 5**. Principal components analysis plots for the first two components for the correlations analysis for 16S rRNA pyrosequencing data from soil (a) and lake sediment (b) data. Numbers in Figure a represent nine different SIP fractions in soil microcosms. 1: Time-point 1, ^12^C ‘heavy’ fraction, 2: Time-point 1, ^13^C ‘heavy’ fraction, 3: Time-point 1, ^13^C ‘light’ fraction, 4: Time-point 2, ^12^C ‘heavy’ fraction, 5: Time-point 2, ^13^C ‘heavy’ fraction, 6: Time-point 2, ^13^C ‘light’ fraction, 7: Time-point 3, ^12^C ‘heavy’ fraction, 8: Time-point 3, ^13^C ‘heavy’ fraction, 9: Time-point 3, ^13^C ‘light’ fraction. Numbers in Figure b represent three different treatments in lake sediment microcosms. 1: ^12^C ‘heavy’ fraction, 2: ^13^C ‘heavy’ fraction, 3: ^13^C ‘light’ fraction. Note that ^13^C ‘heavy’ fraction samples represented with number 2, 5, 8 in Figure (a) and with number 2 in Figure (b) group together.


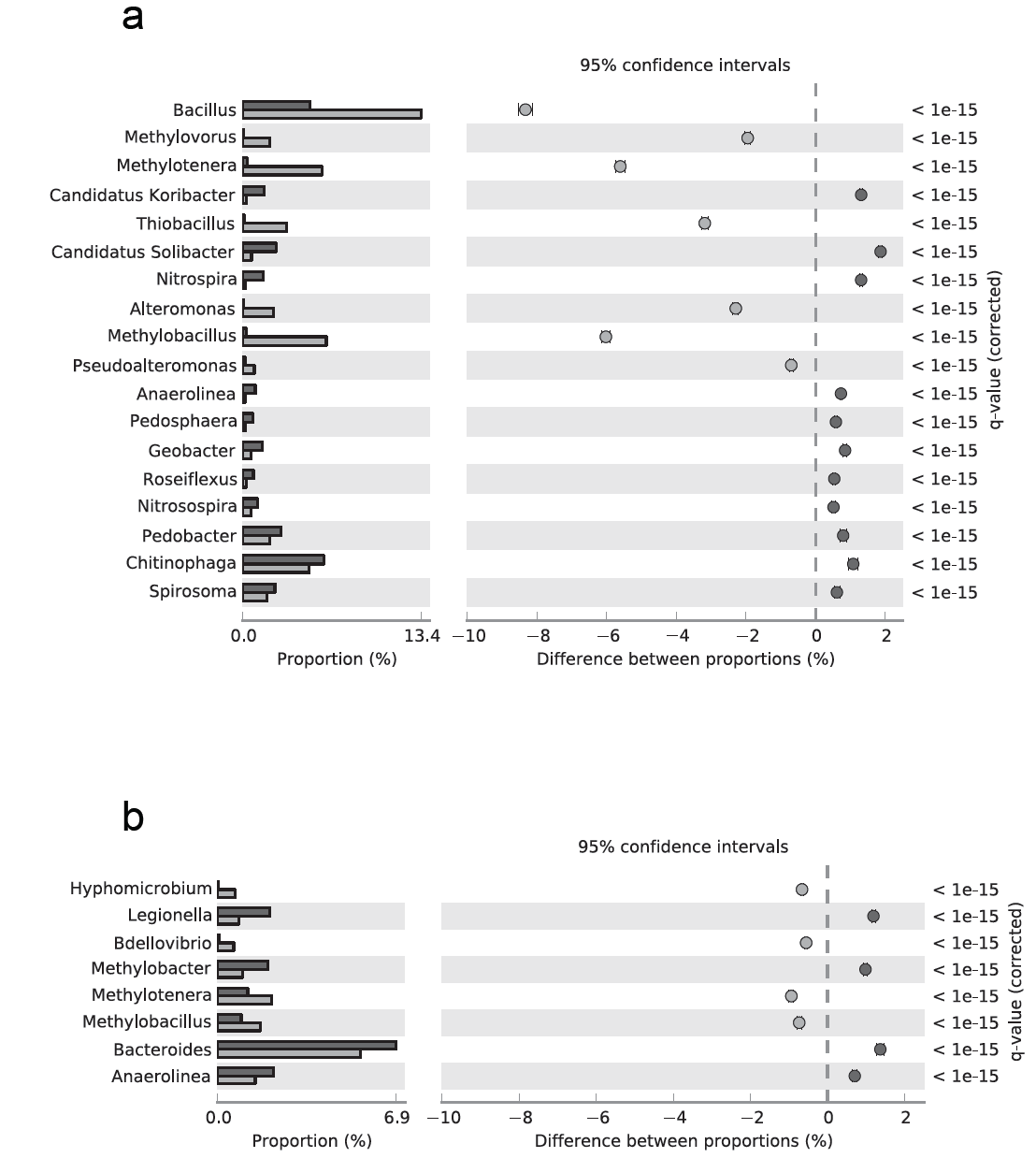


**Supplementary Figure 6**. Genus-level taxonomic analysis of metagenomic sequences obtained from ‘light’ and ‘heavy’ DNA samples from the SIP incubations using the STAMP software. (a) Soil, (b) Lake sediment. Dark grey bars represent the ‘light’ DNA, light grey bars represent the ‘heavy’ DNA.

**Supplementary Figure 7**. Genus-level taxonomic analysis of unassembled reads obtained from ‘heavy’ and ‘light’ DNA samples from the SIP incubations using Kraken. (a) Soil, (b) Lake sediment. Only genera >1% abundance in classified reads are shown.

**
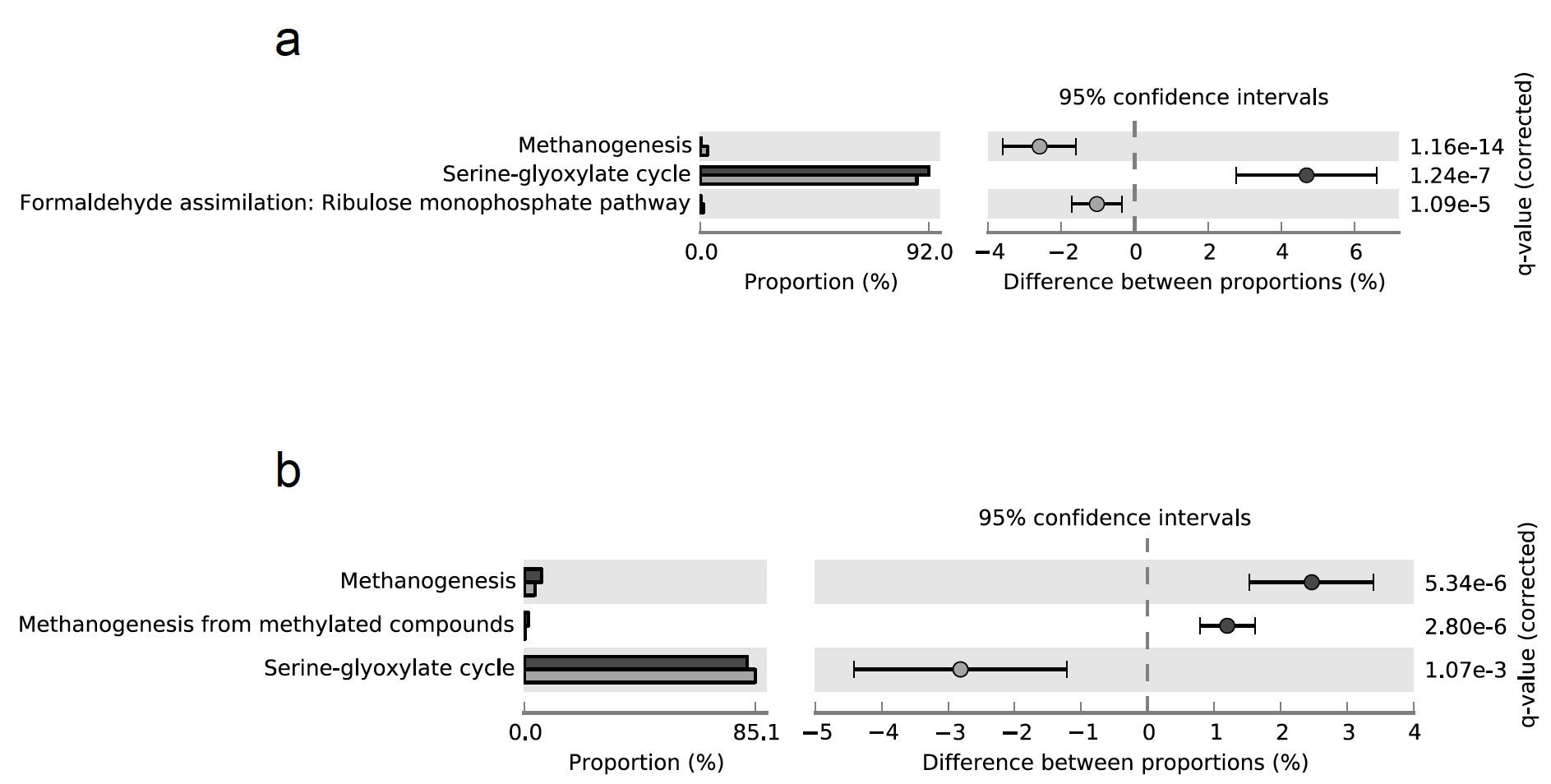
**

**Supplementary Figure 8**. Functional analysis of metagenomic sequences from the one-carbon metabolism category in MGRAST obtained from ‘light’ and ‘heavy’ DNA samples from the soil SIP incubations using the STAMP software. (a) Soil, (b) Lake sediment. Dark grey bars represent the ‘light’ DNA, light grey bars represent the ‘heavy’ DNA.

**
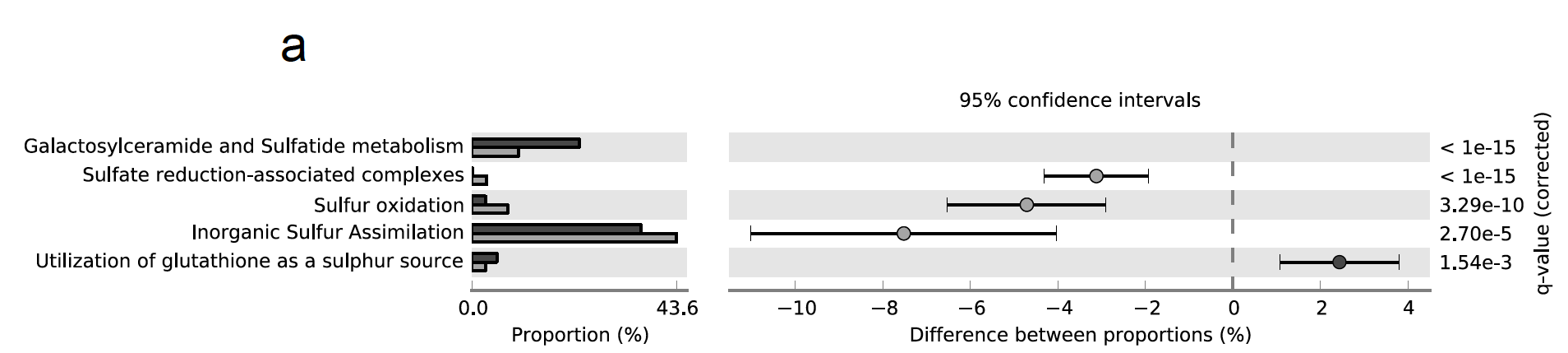
**

**
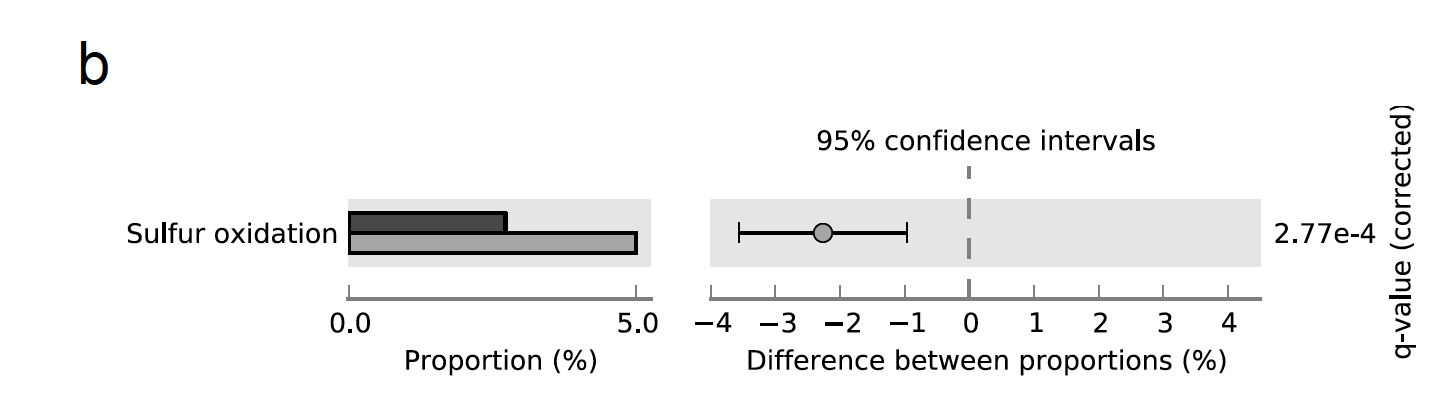
**

**Supplementary Figure 9**. Functional analysis of metagenomic sequences from the ‘Sulfur metabolism’ category obtained from ‘light’ and ‘heavy’ DNA samples using the STAMP software. (a) Soil, (b) Lake sediment. Dark grey bars represent the ‘light’ DNA, light grey bars represent the ‘heavy’ DNA.

**Supplementary Table 1.** Taxonomic classification of the 16S rRNA sequences of the dominant DGGE bands obtained from the DMS-uptake incubations and the second time-point of the lake sediment SIP incubations. Bands were re-amplified, sequenced and analysed by RDP classifier (Wang *et al*., 2007).

**
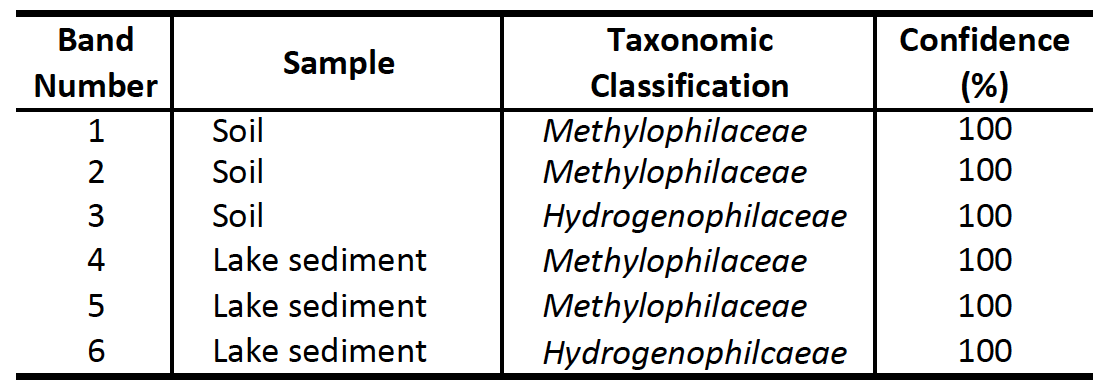
**

**Supplementary Table 3.** Overview of classification of unassembled read data from the soil and lake sediment SIP samples using Kraken.

**
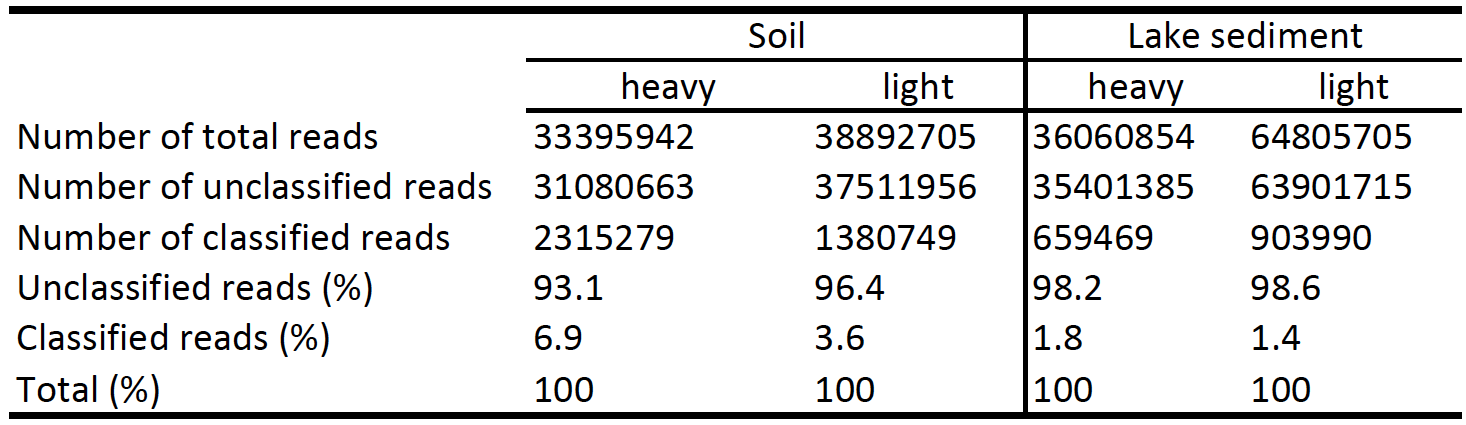
**

**References**

Agresti A (1990). Categorical data analysis. New York: Wiley.

Altschul SF, Gish W, Miller W, Myers EW, Lipman DJ (1990). Basic local alignment search tool. *J. Mol. Biol* **215**:403-410.

Benjamini Y, Hochberg Y (1995). Controlling the false discovery rate: a practical and powerful approach to multiple testing. *J Roy Stat Soc B* **57:**289-300.

Bragg L, Stone G, Imelfort M, Hugenholtz P *et al* (2012). Fast, accurate error-correction of amplicon pyrosequences using Acacia. *Nature Methods* **9**: 425-426.

Caporaso JG, Kuczynski J, Stombaugh J, Bittinger K *et al* (2010a). QIIME allows analysis of high-throughput community sequencing data. *Nature Methods* **7**: 335-336.

Caporaso JG, Bittinger K, Bushman, FD, DeSantis TZ, Andersen GL *et al* (2010b). PyNAST: a flexible tool for aligning sequences to a template alignment. *Bioinformatics* **26**: 266-267.

DeSantis TZ, Hugenholtz P, Larsen N, Rojas M *et al* (2006). Greengenes, a chimera-checked 16S rRNA gene database and workbench compatible with ARB. *App Environ Microbiol* **72**: 5069-5072.

Edgar, RC (2010). Search and clustering orders of magnitude faster than BLAST. *Bioinformatics* **26**: 2460-2461.

Haas BJ, Gevers D, Earl AM, Feldgarden M, Ward DV et al (2011). Chimeric 16S rRNA sequence formation and detection in Sanger and 454-pyrosequenced PCR amplicons. *Genome Res* **21**: 494-504.

Hunter S, Corbett M, Denise H, Fraser M, Gonzalez-Beltran A *et al* (2014). EBI metagenomics - a new resource for the analysis and archiving of metagenomic data. *Nucleic Acids Res* **42**: D600-D606.

McDonald D, Price MN, Goodrich J, Nawrocki EP, DeSantis TZ et al (2012). An improved Greengenes taxonomy with explicit ranks for ecological and evolutionary analyses of bacteria and archaea. *ISME J* 6: 610-618.

Meyer F, Paarmann D, D'Souza M, Olson R, Glass E, Kubal M *et al* (2008). The metagenomics RAST server - a public resource for the automatic phylogenetic and functional analysis of metagenomes. *BMC Bioinformatics* **9:** 386.

Muyzer G, Brinkhoff T, Nübel U, Santegoeds C, Schäfer H, Waver C (1998). Denaturing gradient gel electrophoresis (DGGE) in microbial ecology. In: Akkermans ADL, van Elsas JD, de Bruijn FJ (eds). *Molecular Microbial Ecology Manual*. Kluwer Academic Publishers: Dordrecht, The Netherlands. pp 1–27.

Newcombe, RG (1998). Interval estimation for the difference between independent proportions: comparison of eleven methods. *Stat Med* **17:**873-890.

Schäfer H, Muyzer G (2001). Denaturing gradient gel electrophoresis in marine microbial ecology. In: Paul J.H. (ed.), *Methods in Microbiology* **30**: 425-468, Academic Press, London.

Wang Q, Garrity, GM, Tiedje JM, Cole JR (2007). Naïve Bayesian classifier for rapid assignment of rRNA sequences into the new bacterial taxonomy. *App Environ Microbiol* **73**: 5261-5267.

Wood DE, Salzberg SL (2014). Kraken: ultrafast metagenomic sequence classification using exact alignments. *Genome Biol* **15**: R46.
